# Abdominal Bloating in the Modern Era: Pathophysiological Mechanisms and Environmental Contributors

**DOI:** 10.3390/biom16091359

**Published:** 2026-09-18

**Authors:** Andrea Piccioni, Noelia Di Bari, Marcello Candelli, Angela Saviano, Alessio Migneco, Antonio Gasbarrini, Francesco Franceschi, Veronica Ojetti

**Affiliations:** 1 Department of Emergency Medicine, Catholic University, 00168 Rome, Italy; andrea.piccioni@policlinicogemelli.it (A.P.); marcello.candelli@policlinicogemelli.it (M.C.); angela.saviano@policlinicogemelli.it (A.S.); antonio.gasbarrini@policlinicogemelli.it (A.G.); francesco.franceschi@policlinicogemelli.it (F.F.); 2 Department of Emergency Medicine, Fondazione Policlinico Universitario Agostino Gemelli-IRCCS, 00168 Rome, Italy; noeliadibari9@gmail.com (N.D.B.); alessio.migneco@policlinicogemelli.it (A.M.); 3 Medical and Surgical Science Department, Fondazione Policlinico Universitario Agostino Gemelli-IRCCS, 00168 Rome, Italy; 4 Internal Medicine, Ospedale San Carlo di Nancy, GVM Care and Research, 00165 Rome, Italy; 5 Internal Medicine, Unicamillus International University, 00131 Rome, Italy

**Keywords:** abdominal bloating, gut microbiota, metabolic disorders, psychological stress, environmental exposure, digestive sensitization

## Abstract

Over the past decades, abdominal bloating has been recognized as a common gastrointestinal symptom that significantly affects the quality of life of patients and represents one of the most frequently reported manifestations of gastrointestinal disorders. Abdominal bloating has traditionally been considered a symptom with underlying functional causes, including psychological stress, metabolic disorders, and consumption of a Western-type diet. However, none of these factors fully explains its prevalence. It has been hypothesized that the high prevalence of abdominal bloating may be explained by an increased vulnerability of the digestive system, driven by alterations in the function of the intestinal barrier, as well as in neuroendocrine regulation and environmental and metabolic factors. This review incorporates recent insights into intestinal fermentation, dysbiosis, chronic low-grade inflammation, and gut–brain axis dysfunctions to propose a unified conceptual framework. In this context, chronic exposure to emerging environmental factors could reduce gastrointestinal resilience and modulate responses to fermentative and visceral stimuli. Potential environmental factors include air pollution, microplastics, and endocrine-disrupting chemicals. Overall, these findings suggest a potential role for environmental factors in the modulation of gastrointestinal physiology, although direct evidence linking environmental exposure to abdominal bloating in humans remains limited.

## 1. Introduction

Abdominal bloating is defined as a subjective sensation of fullness or pressure, distinct from abdominal distension, which is characterized by an objectively measurable increase in abdominal circumference [1]. Although they may coexist, abdominal bloating and distension may also occur independently and may be associated with various disorders of gut–brain interaction, including irritable bowel syndrome, functional constipation, and functional dyspepsia [2].

**Figure 1:**
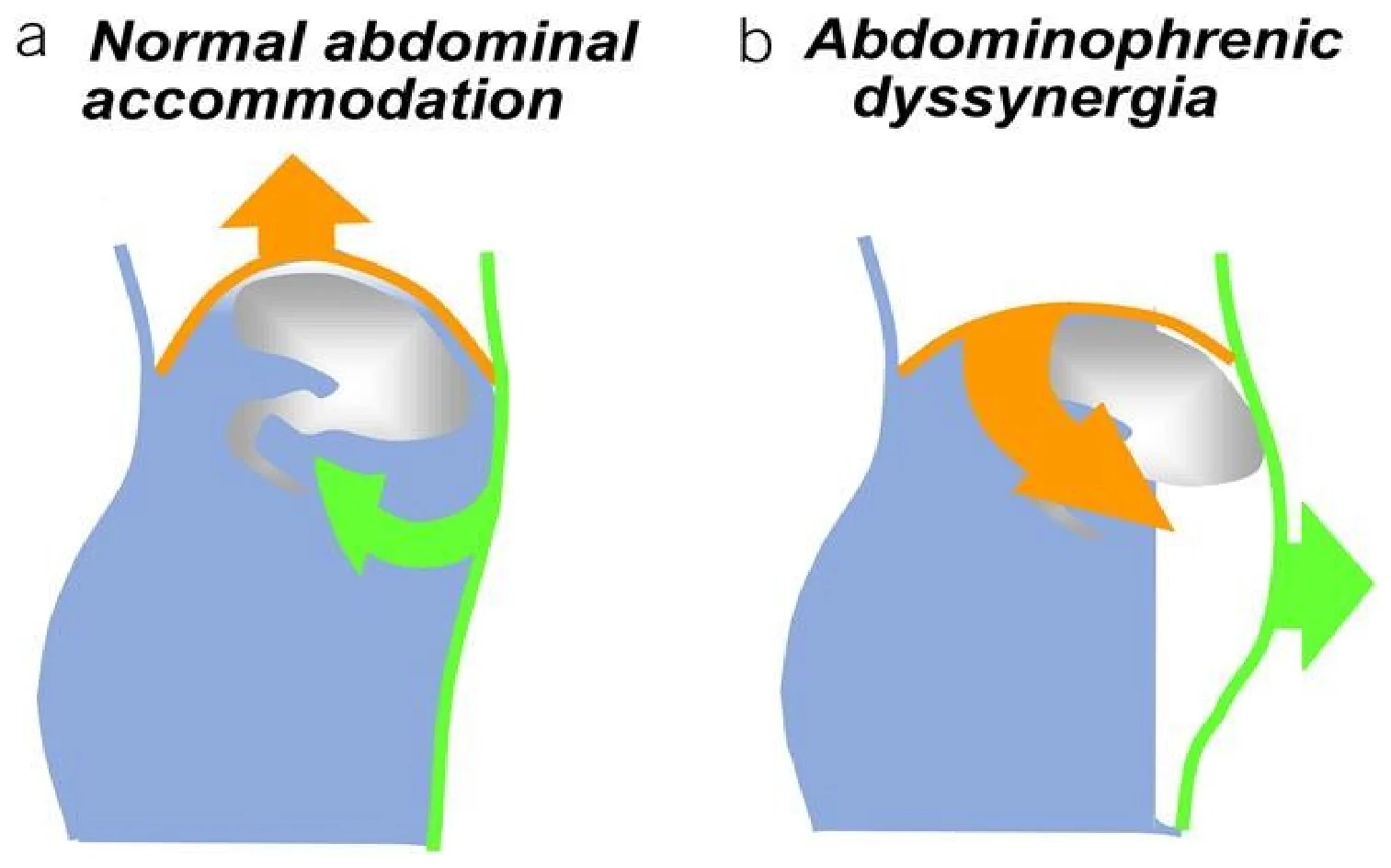
Abdomino-phrenic interactions: (**a**) the diaphragm and abdominal wall coordinate to accommodate abdominal contents without visible protrusion. (**b**) a paradoxical pattern is observed, in which the anterior abdominal wall relaxes while the diaphragm contracts, leading to the appearance of bloating and gas accumulation. Reproduced from [3], under a Creative Commons Attribution 4.0 (CC BY 4.0) license.

Functional abdominal bloating is thought to be primarily associated with visceral hypersensitivity, whereas abdominal distension appears to reflect a somatic and behavioral response associated with abdomino-phrenic dyssynergia, characterized by paradoxical diaphragmatic contraction and relaxation of the anterior abdominal wall (Figure 1) [3]. In patients with functional abdominal bloating, visceral hypersensitivity may lead to an increased perception of physiological intestinal stimuli, such as gas or luminal distension, resulting in discomfort or fullness [4]. This heightened sensitivity may be associated with alterations in peripheral signaling, epithelial barrier function, and gut microbiota composition [2]. Physiological intestinal stimuli may also contribute to abnormal viscerosomatic responses, including abdominophrenic dyssynergia [3,5]. Visceral hypersensitivity therefore represents a core mechanistic driver linking gut sensory dysfunction with the clinical manifestation of functional bloating [1].

In normal circumstances, the physiological compensatory response is characterized by diaphragmatic relaxation and contraction of the anterior abdominal wall. In abdominophrenic dyssynergia, however, there is a dysfunctional neuromuscular pattern in which patients exhibit a paradoxical combination of diaphragmatic contraction and abdominal wall relaxation [3]. This maladaptive somatic behavior increases the anterior protrusion of the abdomen despite minimal or no change in intra-abdominal gas volume. Studies using electromyography and imaging have demonstrated this dyssynergic pattern in patients with functional abdominal distension [6]. Abdominophrenic dyssynergia therefore represents a key mechanistic link between subjective bloating and objective distension [3,6].

Today, these conditions are classified as Disorders of Gut–Brain Interaction (DGBI) and present with persistent or recurrent symptoms in the absence of demonstrable structural or biochemical abnormalities [7]. The lack of objective markers therefore requires assessment based on shared clinical criteria, developed from 1990 onward and progressively systematized in the Rome IV criteria, to ensure nosographic uniformity and reduce the use of inappropriate diagnostic tests [8].

Under physiological conditions, the gastrointestinal tract ensures the elimination of waste products, as well as digestion and nutrient absorption. When these processes are disrupted, reduced absorption of macro- and micronutrients may occur, with consequent effects on nutritional status [9]. Implicated etiopathogenetic factors include alterations of the gut–brain axis, often associated with intestinal microbiota dysbiosis, and dysregulation of the bidirectional gut–brain communication, which may contribute to altered sensory processing and visceral hypersensitivity [10]. Moreover, changes in the gut microbiome may influence intestinal barrier function, microbial metabolism, and gas production, potentially contributing to abdominal bloating [2].

The main mechanisms include abnormal gas handling, abdomino-phrenic dyssynergia, altered gastrointestinal motility, and visceral hypersensitivity [2,3]. Bloating may occur in the absence of evident organic alterations and may be associated with postprandial discomfort, potentially affecting eating behaviors [11]. Given the complexity of these mechanisms, assessing the prevalence of functional bloating and its associated demographic characteristics and risk factors is important.

## 2. Methods

A narrative literature review was conducted to analyze the main pathophysiological mechanisms underlying abdominal bloating and its associated factors, with particular attention to the interactions among the gut microbiota, metabolic determinants, and environmental exposures. The bibliographic search was conducted using major scientific databases, including PubMed, Scopus, and Web of Science. Articles published in indexed, peer-reviewed journals were considered.

The following keywords were used individually or in combination with Boolean operators (AND, OR): “abdominal bloating,” “functional abdominal bloating,” “gut–brain axis,” “gut microbiota,” “visceral hypersensitivity,” “intestinal fermentation,” “air pollution,” “microplastics,” “endocrine disruptors,” and “environmental exposure.”

Original articles, systematic reviews, narrative reviews, and consensus papers published in English were included, with priority given to studies conducted in humans. The search was restricted to the period from 2021 to 2026 to focus on recent evidence in the physiological, clinical, and environmental fields. Preclinical studies with limited translational relevance were excluded. However, selected experimental studies were considered when necessary to provide mechanistic context for emerging environmental exposures.

A large number of articles were initially screened based on the relevance of their titles and abstracts. After full-text evaluation, the studies considered most relevant to the objectives of the review were selected according to their methodological quality, recency, and relevance to the proposed integrative framework. Given the narrative nature of this review, no formal quantitative synthesis or meta-analysis was performed.

## 3. Epidemiology

Estimates of the prevalence of disorders of gut–brain interaction (DGBIs) require careful consideration of the diagnostic criteria used to identify affected individuals, as well as their respective strengths and limitations [12]. The introduction of standardized diagnostic tools, such as the Rome III and the more recent Rome IV criteria, has improved the consistency of DGBI classification [13]. Studies applying the Rome IV criteria have generally reported lower prevalence estimates than those based on Rome III, reflecting more standardized and restrictive diagnostic definitions [14]. Prevalence estimates may also vary according to temporal and geographic context, particularly in settings where awareness and recognition of functional gastrointestinal disorders differ [15].

Large-scale epidemiological studies have reported that DGBIs affect a substantial proportion of the general population, with an overall prevalence of approximately 40% [16]. DGBIs are also associated with a considerable impact on quality of life and healthcare use [16,17]. Women consistently show a higher prevalence of DGBIs than men, with reported odds ratios of 1.7 in an online survey and 1.3 in a household survey [16]. In Italy, approximately 44.2% of adults meet the criteria for at least one DGBI, with a higher prevalence reported among young adults aged 18–39 years, women, and individuals living in Southern Italy [18] (Table 1).

## 4. Diet and Microbiota

The gut microbiota comprises a diverse community of microorganisms involved in immune regulation, intestinal barrier integrity, energy extraction, and protection against pathogens [20]. An imbalance in the gut microbiota, known as dysbiosis, is characterized by alterations in microbial composition and function, including changes in beneficial microorganisms and the expansion of potentially harmful taxa [21]. Diet is one of the main factors influencing the gut microbiota, and dietary patterns characterized by a high consumption of ultra-processed foods and added sugars have been associated with intestinal inflammation and metabolic dysfunction [22].

The interplay between diet and the gut microbiota represents a fundamental concept in gastrointestinal health. Fermentable carbohydrates and dietary fibers can modulate microbial fermentation and the production of metabolites such as short-chain fatty acids (SCFAs) [23,24]. Although these metabolites generally protect intestinal mucosal function, they may negatively influence gas production and intestinal distension in susceptible individuals, thus contributing to abdominal bloating [2]. These processes may also be influenced by environmental factors, dietary habits, and host-related factors, reflecting the complex interplay between the gut microbiota and the host [25].

Ultra-processed foods contain emulsifiers (e.g., carboxymethylcellulose and polysorbate-80) and non-nutritive sweeteners that may alter microbial composition, potentially affecting beneficial and gas-producing taxa [26,27]. These additives may affect tight junctions and the mucus layer, thereby potentially increasing intestinal permeability. This may allow luminal antigens and lipopolysaccharide (LPS) to activate immune pathways and contribute to visceral hypersensitivity, potentially causing physiological gas volumes to be perceived as abdominal bloating. Importantly, increased gas production and abnormal gas perception represent two distinct but potentially interacting processes [1,26]. Moreover, ultra-processed foods have a simplified matrix characterized by low fiber content and a high proportion of rapidly absorbable carbohydrates. This may reduce SCFA production and alter fermentation dynamics, potentially increasing intestinal gas load [23,24].

Disorders of gut–brain interaction (DGBIs) often lead to restrictive dietary patterns, which may contribute to inadequate caloric intake [28]. Among the factors identified in recent years is carbohydrate malabsorption, which may contribute to symptoms such as diarrhea, abdominal pain, and distension. In irritable bowel syndrome (IBS), bloating is particularly common and may be related to alterations in intestinal transit and fermentation [28,29].

To reduce symptom burden, a low-FODMAP diet (fermentable oligosaccharides, disaccharides, monosaccharides, and polyols) is currently recommended, particularly in patients with IBS, as it enables the assessment of individual tolerance to short-chain carbohydrates. FODMAPs include fructans, lactose, fructose, and polyols; these compounds are rapidly fermented, which may increase gas production, including hydrogen and methane, and contribute to abdominal distension. The low-FODMAP approach may modulate intestinal permeability, visceral hypersensitivity, and the production of short-chain fatty acids (SCFAs) [28,29]. It is structured into three phases: an initial restrictive phase (4–8 weeks), an intermediate phase involving the gradual reintroduction of FODMAPs, and a final personalization phase [30]. Considerable interindividual variability has been reported, along with potential alterations in the gut microbiota, nutritional deficiencies, and constipation [31,32]. Moreover, the increased osmotic load associated with FODMAPs may contribute to abdominal distension [31].

**Figure 2:**
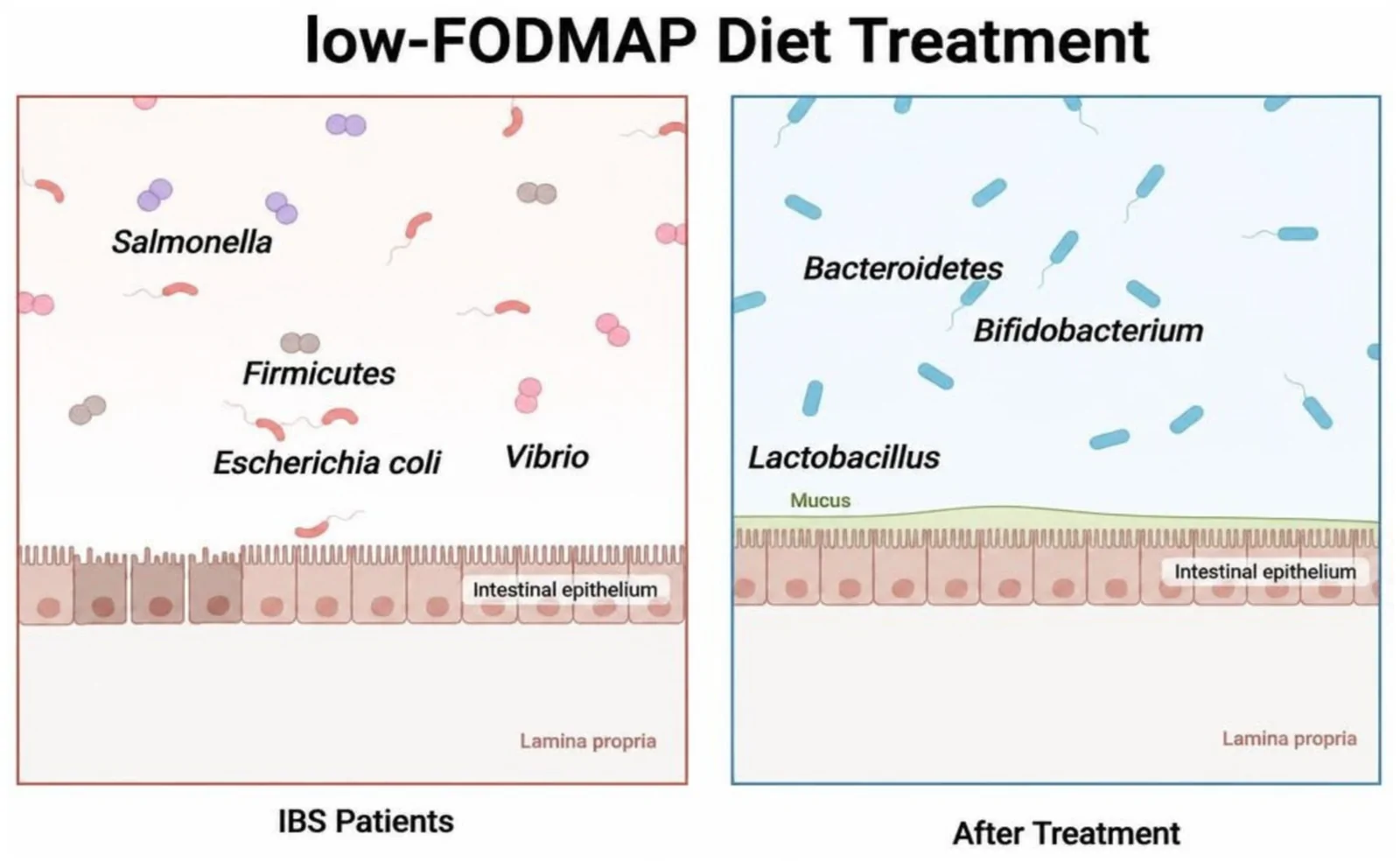
Effect of a low-FODMAP diet on gut microbiota composition, including changes in the relative abundance of beneficial and potentially harmful bacterial taxa. Reproduced from [31], under a Creative Commons Attribution 4.0 (CC BY 4.0) license.

Recent evidence suggests that a low-FODMAP diet may reduce beneficial microorganisms, particularly *Bifidobacterium*, which plays a role in pH regulation and immune modulation. These findings suggest the need for more balanced and sustainable long-term strategies [31,32] (Figure 2).

### Impact of Diet on Fermentation and Intestinal Health

Following digestion by the small intestine, undigested dietary fibers reach the colon, where they are fermented by resident bacteria, leading to the production of gases and metabolites, such as short-chain fatty acids (SCFAs). SCFAs—particularly acetate, propionate, and butyrate—serve as important energy substrates for intestinal epithelial cells and contribute to mucosal homeostasis [33]. Dietary patterns exert a profound influence on gut microbiota composition and metabolic output. Comparative evidence demonstrates differences in the gut microbial profiles between patients following a Mediterranean diet and those following a Western diet. Western-type diets, which are characterized by a high consumption of sugars and ultra-processed foods, were shown to be associated with an unfavorable microbial profile [34].

**Figure 3:**
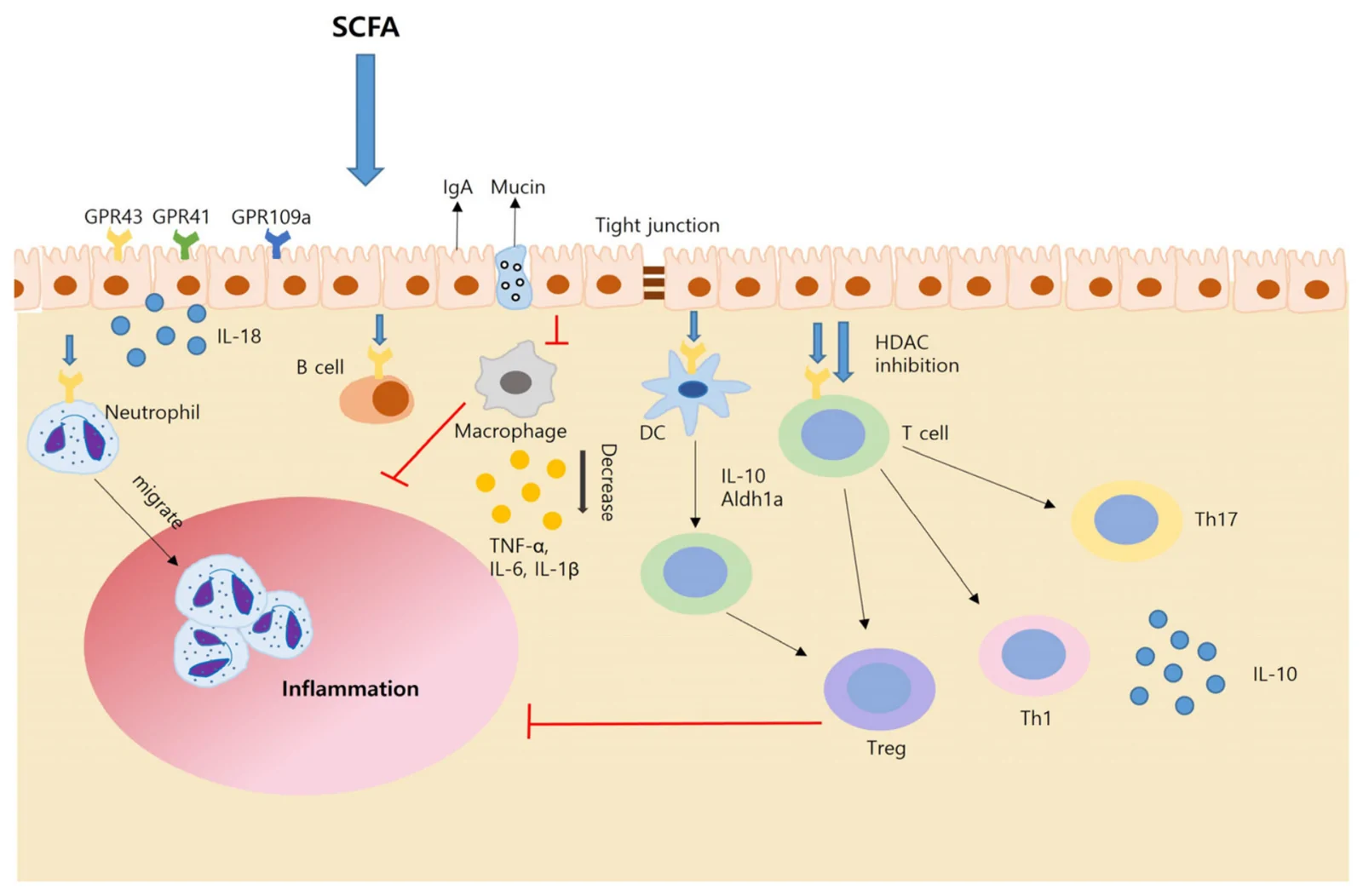
Role of short-chain fatty acids (SCFAs) in immune regulation and inflammatory responses. Reproduced from [33], under a Creative Commons Attribution 4.0 (CC BY 4.0) license.

On the other hand, the Mediterranean diet, which is characterized by a high intake of dietary fiber, polyphenols, and unsaturated fats, has been associated with increased microbial diversity and the presence of beneficial taxa such as *Faecalibacterium prausnitzii*, *Roseburia*, and other butyrate-producing species [35]. These microbes may contribute to mucosal integrity and exert anti-inflammatory effects through short-chain fatty acid production [33,35]. In contrast, the Western diet—typically low in fiber and enriched in refined sugars, saturated fats, and ultra-processed foods—has been associated with reduced microbial diversity and alterations in microbial composition and function [34]. Together, these opposing dietary patterns illustrate how nutrient quality and food matrix complexity may shape gut microbial ecology and contribute to host metabolic and gastrointestinal health [34,35]. Figure 3 summarizes the role of SCFAs in intestinal immune regulation and inflammatory responses, which may contribute to intestinal homeostasis and gastrointestinal symptoms [33].

High-fat diets have been shown to promote the production of endotoxins (lipopolysaccharides) by the microbiota, stimulating Gram-negative bacteria such as *Enterobacteriaceae*, thereby increasing inflammation, obesity, and the risk of cardiovascular diseases [31,32].

Overall, diet plays a crucial role in the regulation of the gut microbiome. Current evidence supports a multifactorial model in the management of bloating, integrating both pharmacological and non-pharmacological treatments, including metabolic, neurophysiological, and behavioral interventions [2].

Pharmacological options, including prokinetics, antispasmodics, gut-directed antibiotics, and neuromodulators, address underlying mechanisms such as impaired motility, visceral hypersensitivity, and dysbiosis [36]. However, optimal symptom control typically requires integration with non-pharmacological interventions [4]. These include structured dietary modification (e.g., Mediterranean-style patterns), psychological therapies targeting gut–brain axis dysfunction, and behavioral approaches such as diaphragmatic breathing or correction of abdominophrenic dyssynergia [3,4]. This combined model reflects the complex, multifactorial pathophysiology of abdominal bloating and aligns with contemporary DGBI management principles. Personalized mechanism-based treatment plans may therefore be appropriate in the management of abdominal bloating given its multifactorial nature [1].

## 5. Metabolism, Inflammation, and Stress

Under physiological conditions, the intestinal barrier plays a crucial role in maintaining the balance between nutrient absorption and protection against potentially harmful luminal factors. Functional alterations of the barrier may increase intestinal permeability, a phenomenon commonly referred to as “leaky gut.” Increased intestinal permeability may contribute to local and systemic inflammatory responses and can be influenced by several factors, including dysbiosis, oxidative stress, autoimmune conditions, and lifestyle-related factors such as alcohol consumption [37].

### 5.1. Mucosal Barrier and Low-Grade Inflammation

The human immune system is a dynamic system whose maturation begins during fetal life and continues throughout development into adulthood [38]. Its function is closely interconnected with the intestinal microbiome and with microbial components such as lipopolysaccharides (LPS) and peptidoglycans. These microbial molecules are recognized by pattern recognition receptors (PRRs), including Toll-like receptors (TLRs) and NOD-like receptors (NLRs). Interactions between the microbiota and the host immune system regulate the production of immunological mediators, including IL-10 and TGF-β, thereby contributing to intestinal homeostasis and local immune regulation [39].

Disruption of intestinal homeostasis may contribute to a persistent inflammatory state known as chronic low-grade inflammation [40]. This condition is characterized by sustained low-level inflammatory activity in the absence of the overt clinical manifestations typically associated with acute inflammation, such as fever. Chronic low-grade inflammation is increasingly recognized as a common mechanism underlying several chronic diseases and may also be influenced by prolonged exposure to environmental factors, including pollutants [41].

### 5.2. Stress and the Gut–Brain Axis

Stress is a key factor influencing intestinal function through the gut–brain axis. The stress response is primarily mediated by activation of the hypothalamic–pituitary–adrenal (HPA) axis, in which corticotropin-releasing factor (CRF) stimulates the release of adrenocorticotropic hormone (ACTH) and subsequently cortisol. Dysregulation of this system has been associated with gastrointestinal symptoms, including bloating and gas, and may contribute to the development or exacerbation of disorders of gut–brain interaction, such as irritable bowel syndrome [42]. Lifestyle factors, including physical activity, sleep, and stress, can influence the gut microbiota and may modulate bidirectional communication between the central nervous system and the gut [43,44]. Physical activity has been associated with changes in gut microbial composition and may increase the abundance of potentially beneficial taxa, including *Faecalibacterium prausnitzii*, *Roseburia*, and *Akkermansia muciniphila* [45]. These microbial changes may influence short-chain fatty acid production and intestinal barrier function [46].

Increased SCFA availability may support anti-inflammatory pathways and contribute to the maintenance of epithelial barrier integrity [37,39,40]. These effects may also influence gut sensory function, although the exact relationship between microbial metabolites, visceral sensitivity, and bloating remains unclear. Physical activity may also influence gastrointestinal function and microbial composition, with potential effects on gastrointestinal motility and gas handling. Collectively, these mechanisms suggest that physical activity may represent a non-pharmacological approach to maintaining microbial and gastrointestinal homeostasis [44,46].

Disrupted or insufficient sleep may alter circadian rhythms within the gastrointestinal tract and the microbiome, potentially favoring changes in microbial composition and function, including SCFA production [47,48]. These alterations may compromise epithelial barrier integrity and contribute to visceral hypersensitivity, potentially modifying the perception of luminal gas [37,40]. Poor sleep may also affect autonomic balance, which could influence gastrointestinal motility and gas retention. Conversely, adequate and regular sleep may support microbial diversity, fermentation dynamics, and gastrointestinal motility, potentially contributing to reduced bloating and improved gastrointestinal comfort [47,48].

Psychological stress is also increasingly recognized as an important modulator of gut microbial ecology and a potential contributor to abdominal bloating. Activation of the HPA axis increases CRH and cortisol levels, which may affect mucosal barrier integrity, gastrointestinal motility, and microbial community structure [49,50]. Experimental evidence indicates that stress can promote gut dysbiosis through CRH–CRHR1-mediated mitochondrial pathways, with an expansion of pro-inflammatory and mucus-degrading taxa and a reduction in beneficial SCFA-producing bacteria [49]. Human and review-based evidence further suggests that biological, environmental, and psychological stressors can modify microbial diversity and composition while affecting intestinal barrier function, immune responses, and gastrointestinal motility [49,50].

From a functional perspective, stress-induced dysbiosis may increase fermentation-related gas production, while stress-related alterations in gut sensory processing may enhance visceral hypersensitivity. Bloating may therefore result not only from changes in luminal gas production but also from altered perception of physiological intestinal distension. These mechanisms are consistent with broader microbiota–gut–brain axis models, in which acute and chronic stress can affect enteric nervous system function, intestinal barrier integrity, and neural signaling, potentially exacerbating bloating and other gastrointestinal symptoms [39,51].

**Figure 4:**
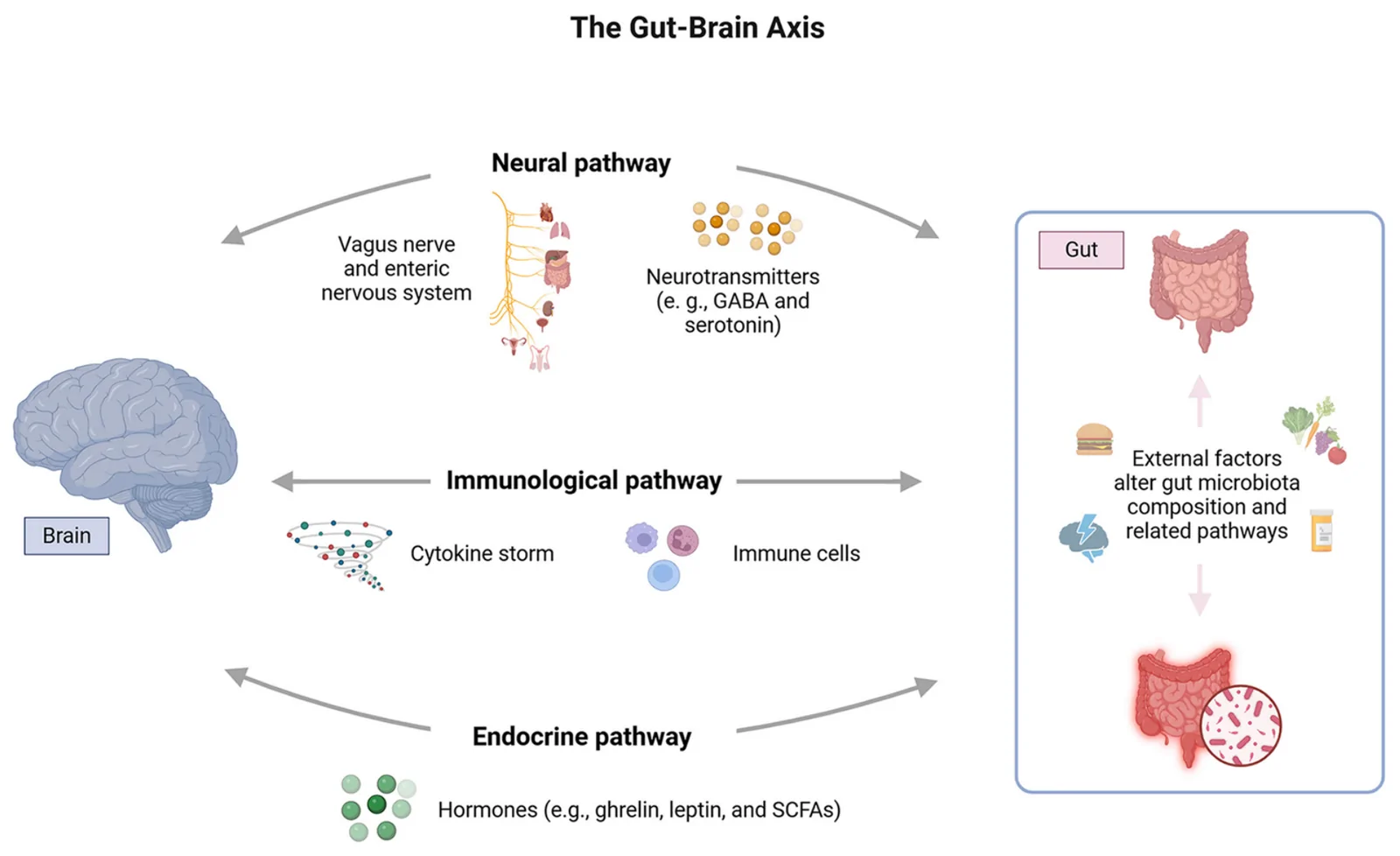
Interactions between the hypothalamic–pituitary–adrenal (HPA) axis and the gut–brain axis (GBA). Reproduced from [55], under a Creative Commons Attribution 4.0 (CC BY 4.0) license.

Taken together, current evidence supports a potential mechanistic relationship between psychological stress, microbiota alterations, and gastrointestinal symptoms. This highlights the potential value of stress-targeted interventions in the management of DGBI. Lifestyle-based approaches, including interventions aimed at modulating the microbiota with probiotics, have therefore attracted increasing interest (Figure 4) [52].

Probiotics may exert strain-specific effects on abdominal bloating and distension through modulation of gut microbial ecology and host mucosal function [53,54]. Certain strains, including *Bifidobacterium infantis* 35624 and *Lactobacillus plantarum* 299V, as well as selected multispecies formulations, have been associated with improvements in bloating and abdominal distension in clinical studies of probiotics in IBS [54]. However, these effects vary according to the specific strain, formulation, and clinical context. Proposed mechanisms include modulation of SCFA production, improvement of epithelial tight-junction integrity, and attenuation of low-grade mucosal inflammation. Probiotics may also influence visceral hypersensitivity through effects on immune signaling and afferent neural pathways. These mechanisms could contribute to changes in luminal distension and symptom perception, supporting the potential role of probiotics as an adjunctive approach in selected patients with gas-related DGBI. Nevertheless, the clinical effects remain strain-specific and should not be generalized across probiotic products [53,54].

The figure summarizes how the HPA and gut–brain axes interact to influence gastrointestinal function and visceral sensitivity. Overall, these processes may contribute to alterations in visceral function and the development of gastrointestinal symptoms. Abdominal bloating should therefore not be considered an isolated symptom, but rather an expression of a complex interaction between intrinsic and extrinsic factors [2]. These factors may predispose individuals to increased reactivity to multiple modulatory stimuli, including environmental conditions.

Although the intestinal system is highly adaptive, exposure to environmental pollutants has been associated with inflammatory responses [56].

## 6. Environmental Factors: Emerging Determinants of Gastrointestinal Sensitization

Air pollution represents an increasingly important environmental factor for human health, particularly in highly exposed areas such as industrialized countries. Exposure to air pollutants has been associated with an increased risk of inflammatory bowel diseases, including Crohn’s disease and ulcerative colitis. Recent studies suggest that these associations may be mediated, at least in part, by epigenetic modifications, particularly DNA methylation, which can alter gene expression and influence individual susceptibility to disease [57,58].

### 6.1. Air Pollution: Microplastics and the Gut Microbiota

**Figure 5:**
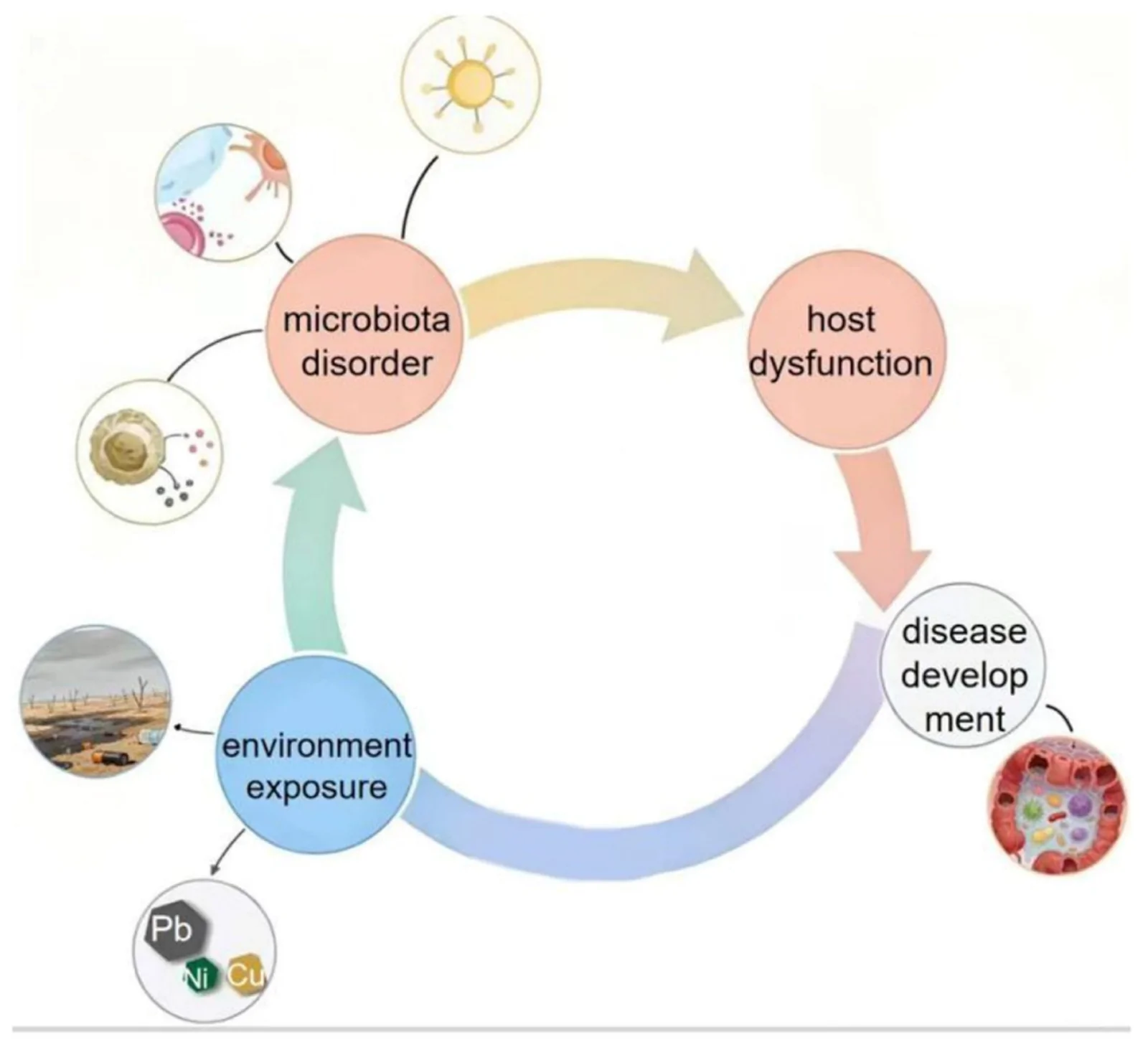
Effects of exposure to environmental pollutants (PM2.5, lead, and POPs) on the gut microbiota. Reproduced from [57] under a Creative Commons Attribution 4.0 (CC BY 4.0) license.

The gut microbiota can be influenced by exposure to atmospheric pollutants through the ingestion of contaminated food and water and through inhalation followed by the swallowing of airborne particles, including microplastics [59]. Environmental contaminants relevant to gut health include air pollutants, heavy metals, persistent organic pollutants (POPs), and emerging contaminants such as pharmaceuticals and personal care products (Figure 5) [57].

Once they reach the intestine, these pollutants may interact with the epithelial barrier, promoting the production of pro-inflammatory mediators and contributing to alterations in microbial community structure [60]. Exposure to heterogeneous mixtures of pollutants, including particulate matter and gases with different physicochemical properties and levels of toxicity [61], may also affect the production of short-chain fatty acids (SCFAs). Alterations in SCFA-producing bacterial taxa have been reported and may have systemic consequences, potentially contributing to immunometabolic and inflammatory imbalance [61,62].

In parallel, the detection of microplastics in the systemic circulation has raised concerns regarding their potential biological effects, including endocrine disruption, oxidative stress, and alterations of the gut microbiota. These effects may have gastrointestinal consequences, although direct evidence linking microplastic exposure to abdominal bloating or distension in humans remains limited [63]. Emerging evidence suggests that microplastics may alter microbial composition, causing a reduction in the level of *Bifidobacterium* and *Lactobacillus* and an increase in gas-producing taxa, such as *Clostridia* and *Enterobacteriaceae*, potentially reducing overall microbial diversity [64,65].

Polystyrene particles, a specific type of microplastic, have been associated with alterations in intestinal barrier integrity and inflammatory responses, including changes in tight-junction proteins, increased intestinal permeability, and oxidative stress. Microplastics may also trigger an increase in pro-inflammatory cytokines, such as IL-6 and TNF-α, as well as macrophage activation. These findings suggest that microplastic exposure may affect gastrointestinal homeostasis, although the link of these effects to visceral hypersensitivity and abdominal bloating in humans remains unclear [63].

### 6.2. Metabolic Effects of Environmental Pollutants on Abdominal Symptoms

Among emerging environmental factors, endocrine-disrupting chemicals represent widely distributed potentially harmful substances in the environment. These compounds can interact with hormone receptors and endocrine pathways, contributing to metabolic alterations with possible intestinal consequences [66].

In this context, micro- and nanoplastics may act as carriers of endocrine disruptors such as bisphenol A and phthalates [67], which, once released, can bind to hormone receptors and affect reproductive and developmental systems. Phthalate exposure has been associated with obesity, impaired glucose regulation, and hypertension [68]. Moreover, due to their small size and high reactivity, experimental studies suggest that nanoplastics may cross the intestinal epithelium, with potential implications for systemic distribution and long-term bioaccumulation. Similarly, certain persistent organic pollutants such as polychlorinated biphenyls (PCBs) and polybrominated biphenyls (PBBs) tend to accumulate in adipose tissue and have been linked to thyroid dysfunction, oxidative stress, and inflammatory processes, contributing to the development of insulin resistance [67,68,69].

Overall, the gut microbiota and its alterations are influenced by multiple intrinsic factors (age, sex) and extrinsic factors (diet, pollution, stress), which contribute to the development of metabolic conditions such as obesity and diabetes. This supports the hypothesis of an integrated axis between environment, metabolism, and intestinal function [69,70].

A relevant aspect to consider is that current evidence suggests that the environment does not act merely as a collection of risk factors, but may progressively contribute to long-term modifications of intestinal physiology and disease manifestations [69]. Although promising, these findings remain limited and require further confirmation through longitudinal clinical studies.

### 6.3. Neuroenteric Dysregulation: Exposome Perspectives

Neuroenteric dysregulation refers to impaired communication between the central nervous system, enteric nervous system, and gut microbiota. In recent years, alterations in gut microbiota composition and function have been increasingly considered in relation to environmental and lifestyle exposures. The concept of the exposome refers to the totality of environmental, dietary, and psychosocial exposures to which an individual is subjected throughout modern life [71]. These exposures may contribute to low-grade inflammation and alterations in neuroimmune signalling, potentially leading to heightened visceral sensitivity and altered gastrointestinal sensory processing [72]. Microbiota-derived signals and metabolites act as key intermediaries in the communication between the gut and the nervous system, influencing gastrointestinal function and sensory processing [73]. Several mechanisms, beyond epithelial and immune alterations, may influence neuroenteric regulation through interactions between the autonomic nervous system, gut microbiome, and environmental exposures, potentially affecting systemic physiology and neurovisceral function [74]. From an exposome perspective, neuroenteric dysregulation is therefore not a single-factor phenomenon but the cumulative result of interacting environmental, behavioral, and biological influences that may affect gut–brain axis function and contribute to bloating and other DGBI symptoms [10].

Current evidence indicates that bloating can result from visceral hypersensitivity, altered motility, impaired gas handling, microbiota-driven fermentation, low-grade inflammation, and maladaptive somatic responses such as abdominophrenic dyssynergia. These mechanisms vary widely between individuals and often coexist, making it difficult to identify one dominant cause [1].

Moreover, environmental and lifestyle factors, including diet, stress, sleep, physical activity, and exposure to ultra-processed foods, may influence the microbiota, adding further variability [75]. The heterogeneity of patient phenotypes and the complexity of the underlying pathophysiological mechanisms contribute to the challenge of fully elucidating bloating and other DGBI symptoms [76].

As a result, bloating is best conceptualized as a multifactorial, systems-level condition, where symptoms emerge from the cumulative interaction of biological and behavioral influences rather than a single definable pathology. Despite advances in the understanding of abdominal bloating, it remains a complex condition that is not yet fully elucidated, involving multiple interrelated factors [1]. The integration of large-scale data may contribute to a more precise understanding of the mechanisms underlying abdominal distension. Further studies are needed to better understand these emerging phenomena and clarify their clinical significance.

## 7. Limitations

This review has several limitations. Most of the available evidence is based on associative studies with limited human data. In addition, the narrative nature of this review may introduce selection bias.

The available literature is heterogeneous in terms of study design, study populations, and outcomes, which may limit the direct comparison of findings and the assessment of the strength of the reported associations. Although priority was given to human studies, some of the mechanisms discussed, particularly those related to environmental exposures, are supported mainly by preclinical or indirect evidence. Therefore, the proposed relationships between environmental exposures, gut microbiota alterations, intestinal barrier function, inflammation, neuroimmune signaling, and abdominal bloating should be interpreted with caution and cannot yet be considered a demonstrated causal pathway in humans. The literature search was limited to studies published between 2021 and 2026, with the aim of focusing on the most recent evidence. While this approach allowed us to focus on recent evidence, it may have excluded some earlier studies that could be relevant to understanding the pathophysiology of abdominal bloating.

Furthermore, the different components of the proposed framework are supported by different levels of evidence. Some mechanisms are relatively well established, whereas others remain preliminary and should be regarded primarily as hypotheses requiring further investigation. Therefore, the proposed integrative framework should be interpreted as a conceptual model that requires further validation through longitudinal clinical and translational studies.

## 8. Conclusions

Abdominal bloating is a common and complex gastrointestinal symptom frequently reported in the general population. Although factors such as diet, psychological stress, ultra-processed food consumption, and genetic predisposition may influence intestinal function, gut–brain axis dysfunction appears to play a central role in its pathophysiology, supporting a therapeutic approach aimed at the patient as a whole rather than at the isolated symptom.

Increasing attention has recently been directed toward environmental changes and external exposures and their potential role in increasing susceptibility to functional gastrointestinal alterations. In particular, a possible involvement has been hypothesized in metabolic, neuroenteric, and inflammatory changes observed in younger populations.

Within this framework, intestinal bloating may represent not only an isolated symptom, often manageable through lifestyle modifications, but also a potential indicator of broader physiological involvement. Emerging evidence suggests a possible association between external factors and gastrointestinal symptomatology, although the potential relationship between environmental exposures and abdominal bloating remains to be established.

Available evidence remains limited and does not yet allow for definitive conclusions. Despite advances in mechanistic understanding, these conditions remain only partially elucidated, with substantial interindividual variability in symptom expression and response to interventions. Emerging evidence suggests that environmental exposures may contribute to gastrointestinal dysfunction through effects on the gut microbiota, epithelial barrier integrity, neuroimmune signaling, and visceral sensitivity. Future research should clarify if such environmental factors act as primary drivers of gastrointestinal symptoms in modern societies, enabling earlier identification of underlying mechanisms and potentially informing strategies to prevent broader systemic effects. To achieve this, studies should test whether pollutant-induced dysbiosis predicts bloating severity, whether early epithelial barrier disruption serves as a biomarker for symptom development, and whether neuroimmune pathway activation mediates exposure-related visceral hypersensitivity. Integrating large-scale exposome, microbiome, and multi-omics datasets will be essential in order to develop a more precise and comprehensive understanding of the pathways that contribute to abdominal distension, and to allow for targeted mechanism-based therapeutic strategies.

## Figures and Tables

**Table 1:** Epidemiological characteristics of disorders of gut–brain interaction and abdominal bloating.

Parameter	Epidemiological Data	Associated Characteristics
Prevalence of DGBIs in the general population	Approximately 40% of the general population [16].	Higher prevalence among women; greater healthcare utilization [16,17].
Prevalence of functional abdominal bloating and distension	Approximately 3.5% of the general population [19].	More frequently reported among women, with a female-to-male ratio of approximately 2:1 [19].
Patients with bloating referred to gastroenterology clinics	Abdominal bloating is reported in approximately 23% of patients referred to a general gastroenterology clinic [19].	Bloating represents a frequent symptom among patients undergoing gastroenterological evaluation [19].
Impact on quality of life	Approximately 75% of patients with bloating describe their symptoms as moderate to severe [19].	Chronic abdominal bloating and distension may substantially impair quality of life [19].

## Data Availability

No new data were created or analyzed in this study. Data sharing is not applicable to this article.
